# Evaluation of Average Quantum Efficiency of Industrial Digital Camera

**DOI:** 10.3390/s25030899

**Published:** 2025-02-02

**Authors:** Zhuochen Deng, Lingfeng Chen, Xuemeng Wei, Xusheng Zhang

**Affiliations:** 1 School of Optics and Photonics, Beijing Institute of Technology, Beijing 100081, China; bit_dzc@163.com (Z.D.); 3220220477@bit.edu.cn (X.W.); zhangxs@bit.edu.cn (X.Z.); 2 National Key Laboratory on Near-Surface Detection, Beijing 100081, China

**Keywords:** quantum efficiency, average quantum efficiency, industrial digital cameras, camera performance evaluation

## Abstract

Quantum efficiency (QE) is a critical metric for assessing the performance of industrial digital cameras. The current EMVA1288 standard relies on monochromatic light for QE measurements. Comprehensive QE tests across the visible spectrum often involve elaborate setups and extensive data acquisition. Additionally, such tests may not fully capture camera performance under broadband illumination, which is frequently encountered in industrial applications. This study introduces the concept of average quantum efficiency (AQE) using white light sources and proposes a novel testing method. Systematic experiments and data analyses were performed on two industrial digital cameras under white light sources with different spectral distributions. The results suggest that AQE testing offers a practical and efficient means to evaluate camera performance under broadband illumination, complementing existing monochromatic QE measurement methods.

## 1. Introduction

Quantum efficiency (QE) is a fundamental parameter that quantifies the ability of a camera to convert incident photons into electrons. It is defined as the ratio of the electrons generated by a pixel to the total number of photon incidents on that pixel during a specific exposure time. QE plays a pivotal role in determining camera performance, influencing not only sensitivity but also key parameters such as the signal-to-noise ratio, dynamic range, and overall image quality [1]. The improvement of QE has been achieved through techniques such as back-illuminated process optimization [2], surface nanostructuring [3], and the development of silicon optical antenna-based color sensors [4]. Studies have extended QE measurements from visible to ultraviolet and X-ray wavelengths, enabling broader applications in biomedical imaging and material analysis [5,6,7].

To standardize testing methods and ensure the consistency of results, the European Machine Vision Association (EMVA) introduced the EMVA 1288 standard [8]. This standard defines the principles and conditions for measuring multiple electro-optical performance parameters of image sensors, including QE, providing a unified framework for the testing process and promoting industry standardization. Traditional QE testing involves monochromators and integrating spheres for uniform illumination, with methods requiring extensive experimental setups and prolonged data acquisition. Sperlich and Stolz [9] presented a method for measuring the spectral quantum efficiency (SQE) of EMCCD cameras, revealing discrepancies with manufacturer data, especially for back-illuminated models with UV conversion layers. They demonstrated temperature-dependent SQE changes, with lower temperatures boosting blue-range SQE and reducing red-range SQE, with invariant points at 710 nm and 740 nm. Recent advancements have explored alternative methods for QE measurement to improve flexibility and efficiency. Junlin Li, et al. [10] employed a dynamic scanning technique with rotating mirrors, offering high efficiency and precision for linear array cameras. Yuri Rzhanov [11] demonstrated a single-image QE determination method, overcoming traditional methods’ dependence on optical stability and extensive calibration.

The current EMVA 1288 standard remains invaluable for ensuring the consistency and precision of QE measurements under controlled monochromatic illumination. However, its focus on monochromatic light sources may not fully represent camera performance under complex lighting environments, such as those encountered in industrial applications involving white or natural light sources.

In this work, we propose a simple method for estimating the average quantum efficiency (AQE) of image sensors under broadband illumination. This approach seeks a complementary perspective by offering practical insights into camera performance under real-world lighting conditions. By using white light sources and introducing the concept of AQE, the method highlights sensor response over a broader spectrum, enhancing the applicability of the results in diverse scenarios.

## 2. Measurement Model of AQE

**Figure 1:**
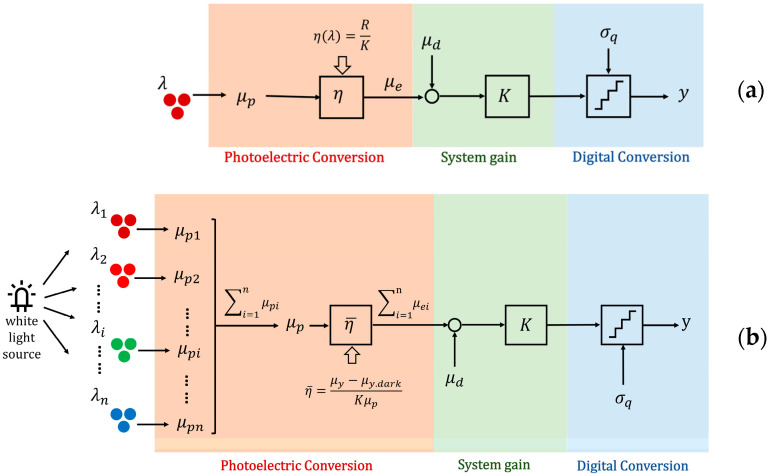
Mathematical model of digital camera: (**a**) monochromatic light source; (**b**) white light source.

The principle of a digital camera is as follows: incoming photons are converted into electrons by the photoelectric effect, which are then transformed into a voltage signal, amplified, and finally converted into digital signal by an analog-to-digital converter. As illustrated in Figure 1, this process can be sequentially divided into three stages: photoelectric conversion, system gain, and digital conversion. While the stages of this process are the same for both monochromatic and white light sources, we will first describe the traditional monochromatic light model for simplicity and clearer understanding. Only then do we introduce the new method for AQE measurement under a white light source, which considers the combined effects of multiple wavelength components to assess camera performance under more realistic lighting conditions.

Figure 1a represents the three stages under monochromatic light. During the exposure time, an average $\mu_{p}$ photon with a wavelength λ hit the whole area of a single pixel, of these, $\mu_{e}$ electrons are accumulated and finally converted into a digital number y. The fraction $\eta \left(\lambda \right)=\mu_{e}/\mu_{p}$ is defined as the quantum efficiency at wavelength λ. According to the EMVA1288 standard [8], the overall system gain is assumed to be linear and can be described by a single quantity K with units DN/e-(digital numbers per electron). The mean number of electrons present without incident light $\mu_{d}$ and an additional noise component $\sigma_{q}$, uniformly distributed within the quantization intervals, contribute to the overall system noise. The QE is calculated by dividing the responsivity R by the overall system gain K (R is the parameter used in the standard to describe the camera’s overall response at wavelength λ, with units DN/p, i.e., digital numbers per photon).

As Figure 1b shows, a white light source is assumed to consist of multiple monochromatic light components with central wavelengths $\lambda_{1},\lambda_{2}\ldots \lambda_{n}$. When white light is incident on the sensor, the total number of photons is denoted as $\mu_{p}$ and the total number of photo-generated electrons is $\mu_{e}$. The AQE $\left(\bar{\eta }\right)\;$is defined as

(1) $$\bar{\eta }=\frac{\mu_{e}}{\mu_{p}},$$

where $\mu_{e}=\sum_{i=1}^{n}\mu_{e_{i}}$, $\mu_{p}=\sum_{i=1}^{n}\mu_{p_{i}}$.

We use the overall system gain *K* to represent the digital numbers per electron under white light illumination, the mean DN $\mu_{y}$ can be expressed as

(2) $$\mu_{y}=K(\mu_{e}+\mu_{d}),$$

where $\mu_{d}$ represents the mean number of dark electrons. Substituting $\mu_{y.dark}=K\mu_{d}$ into Equation (2), we get

(3) $$\mu_{y}-\mu_{y.dark}=K\mu_{e}.$$

Substituting Equation (3) into Equation (1),

(4) $$\bar{\eta }=\frac{\mu_{y}-\mu_{y.dark}}{K\mu_{p}}\;.$$

To calculate $\bar{\eta }$, it is necessary to obtain the corresponding values for three parameters: (1) The mean photo-induced DN$\;\left(\mu_{y}-\mu_{y.dark}\right)$; (2) the overall system gain *K*; and (3) the number of incident photons $\mu_{p}$.

### 2.1. Parameter 1: Mean Photo-Induced DN $\left(\mu_{y}-\mu_{y.dark}\right)$

The mean of the DN $\left(\mu_{y}-\mu_{y.dark}\right)$ over all pixels in the active area at each exposure time is computed from two consecutively captured *M* × *N* images $y^{A}$ and $y^{B}\;$as

(5) $$\mu_{y}=\frac{1}{2NM}\sum_{m=0}^{M-1}\sum_{n=0}^{N-1}\left(y^{A}\left[m\right]\left[n\right]+y^{B}\left[m\right]\left[n\right]\right),$$

averaging over *M* rows and *N* columns. $y^{A}\left[m\right]\left[n\right]$ denotes the DN of the pixel at row m, column n in image $y^{A}$, which is similar to $y^{B}\left[m\right]\left[n\right]$.

In the same way, the mean DN of dark images $\mu_{y.dark}$, can be calculated from two consecutive dark-field images, $y^{A.dark}$ and $y^{B.dark}$, as

(6) $$\mu_{y.dark}=\frac{1}{2NM}\sum_{m=0}^{M-1}\sum_{n=0}^{N-1}\left(y^{A.dark}\left[m\right]\left[n\right]+y^{B.dark}\left[m\right]\left[n\right]\right),$$

the notation for $y^{A.dark}\left[m\right]\left[n\right]$ and $y^{B.dark}\left[m\right]\left[n\right]$ follows the same convention as $y^{A}\left[m\right]\left[n\right]$ and $y^{B}\left[m\right]\left[n\right]$.

### 2.2. Parameter 2: Overall System Gain K

According to the Poisson distribution theory, the variance of charge fluctuations is equal to the average number of accumulated charges, as given by

(7) $$\sigma_{e}^{2}=\mu_{e}.$$

This noise, often referred to as shot noise, is given by the basic laws of physics and applies to all types of cameras.

From Equation (3), the variance of the photoelectrons $\sigma_{e}^{2}$ is related to the variance of the output DN $\sigma_{y}^{2}$ and the variance of the dark-field DN $\sigma_{y.dark}^{2}$ as follows

(8) $$\sigma_{y}^{2}-\sigma_{y.dark}^{2}=K^{2}\sigma_{e}^{2}.$$

Substituting Equation (7) into Equation (8), we obtain

(9) $$\sigma_{y}^{2}-\sigma_{y.dark}^{2}=K\cdot K\mu_{e}=K\cdot (\mu_{y}-\mu_{y.dark}).$$

Equation (9) shows that the overall system gain *K* can be determined from the slope of the linear relation between the variance of the noise ${(\sigma }_{y}^{2}-\sigma_{y.dark}^{2})$ and the mean photo-induced DN $\left(\mu_{y}-\mu_{y.dark}\right)$. The variance of the noise ${(\sigma }_{y}^{2}-\sigma_{y.dark}^{2})$ can be calculated with Equations (10) and (11)

(10) $$\sigma_{y}^{2}=\frac{1}{2NM}\sum_{m=0}^{M-1}\sum_{n=0}^{N-1}{\left(y^{A}\left[m\right]\left[n\right]-y^{B}\left[m\right]\left[n\right]\right)}^{2},$$

(11) $$\sigma_{y.dark}^{2}=\frac{1}{2NM}\sum_{m=0}^{M-1}\sum_{n=0}^{N-1}{\left(y^{A.dark}\left[m\right]\left[n\right]-y^{B.dark}\left[m\right]\left[n\right]\right)}^{2}.$$

### 2.3. Parameter 3: Number of Incident Photons $\mu_{p}$

According to the EMVA1288 standard, the mean number of incident photons $\mu_{p_{i}}$ of wavelength $\lambda_{i}$ that hit a pixel with the area A during the exposure time $t_{exp}$ can be computed from the irradiance $E_{i}$ on the sensor surface and the quantization of the energy of electromagnetic radiation $h\nu_{i}$ as

(12) $$\mu_{p_{i}}=\frac{A{\cdot E}_{i}{\cdot t}_{exp}}{h\cdot \nu_{i}}=\frac{A\cdot E_{i}\cdot t_{exp}}{h\cdot c/\lambda_{i}},$$

where the speed of light *c* = 2.99792458 × 10⁸ m/s and Planck’s constant h = 6.6260755 × 10⁻³⁴ J·s. When using the common units for sensor parameters ($A:\;{\mu m}^{2},\;t_{\exp}:\;\mathrm{ms},\;\lambda_{i}:\;\mu m,E_{i}:\;\mu W/{cm}^{2}$), the equation becomes

(13) $$\mu_{p_{i}}=50.34\cdot A\cdot t_{exp}\cdot \lambda_{i}\cdot E_{i}.$$

Equation (13) applies to a monochromatic light source. When using a white light source, the total number of photons $\mu_{p}$ should be calculated using the spectral irradiance of the light source (with units of $\mu W/{cm}^{2}/nm$) as follows

(14) $$\mu_{p}=\sum_{i=1}^{n}\mu_{p_{i}}=50.34\cdot A\cdot t_{exp}\cdot \sum_{i=1}^{n}\left(\frac{\lambda_{i}+\lambda_{i+1}}{2}\cdot \frac{E_{i}+E_{i+1}}{2}\cdot (\lambda_{i+1}-\lambda_{i})\right),$$

where$\;\lambda_{i}$ represents the starting wavelength of the *i*-th interval in a discretized range of n intervals. The center wavelength of the interval is given by ${(\lambda }_{i}+\lambda_{i+1})/2$, and $E_{i}$ represents the irradiance value corresponding to the wavelength $\lambda_{i}$ in the spectral irradiance data.

## 3. Experiments of AQE Measurement

### 3.1. Overall Structure of the Testing System

**Figure 2:**
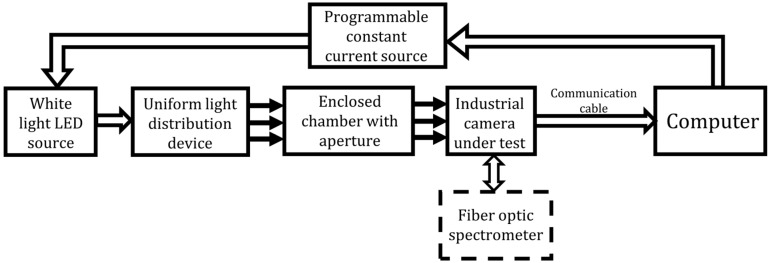
Overall structure of the testing system.

The testing system’s structure is illustrated in Figure 2. It comprises a white light LED source, a programmable constant current source, a uniform light distribution device, an enclosed chamber with an aperture, an optical fiber spectrometer, the industrial camera under test, and a computer. The LED light source is processed to produce a uniform surface light source with a diameter D, which is fixed at the inlet of the chamber, while the camera is positioned at the chamber’s outlet. The distance d between the image sensor and the surface light source satisfies the condition d = 8 × D. The optical fiber spectrometer measures the spectral irradiance distribution of the light source at the image sensor’s position. The computer controls the operation of the entire testing system and simultaneously collects and processes spectral irradiance and image data.

**Figure 3:**
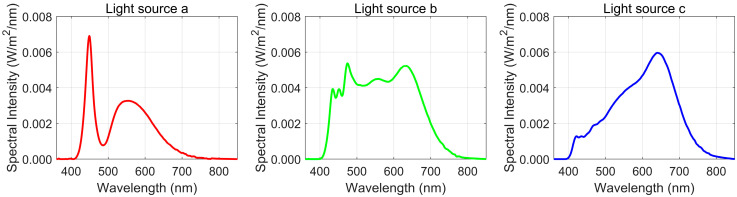
Spectral irradiance distributions of light sources a − c.

Three white light LED sources with distinct spectral characteristics were selected for the experiment. Figure 3 presents their measured spectral irradiance curves. Light source a is a blue-light-excited white LED with a peak near 450 nm, a correlated color temperature (CCT) of approximately 6616 K, a color rendering index (CRI) $R_{a}$ of 75, and a Deviation from the Planckian Locus in the Uniform Color Space (Duv) value of 5.3 × 10^−4^. Light source b is a daylight-simulating LED, featuring a relatively flat spectrum across the visible range, with a CCT of around 5143 K and a CRI $R_{a}$ of 94, a Duv value of −6.1 × 10^−4^. Light source c exhibits peak power at 650 nm and has a CCT of approximately 3335 K, with a CRI $R_{a}\;$of 98 and a Duv value of 2.2 × 10^−3^.

Two digital cameras were selected as test samples for the experiment; Table 1 summarizes their key parameters. The optical fiber spectrometer used in this work is the Ocean Optics HR4000CG UV-NIR, which covers a wavelength range from 200 to 1100 nm with a spectral resolution of 0.75 nm. The spectrometer operated in absolute irradiance mode during the experiment.

### 3.2. Testing Process and Results

The testing process adheres to the variable illumination method specified in the EMVA1288 standard. The LED white light source is fixed at the chamber’s inlet, while the industrial camera under test is positioned at the outlet. The camera’s exposure time is set to 2.5 ms for Camera A and 1.61 ms for Camera B, and the constant current source is adjusted to vary the LED’s illumination. A minimum of 25 equally spaced illumination levels are used, covering a range from the dark DN to the maximum DN.

After image acquisition, the camera is removed from the outlet of the chamber, and the optical fiber spectrometer is replaced to measure the spectral irradiance distribution of the light source at the image sensor’s position, the data are essential for determining the irradiance values that contribute to the AQE calculations in Section 2. For the C-mount industrial camera, the spectrometer’s probe is positioned 17.5 mm from the chamber outlet. The camera is then reinstalled to continue capturing digital images.

Two digital images are saved for each current setting, yielding a total of 50 bright-field images. For dark image acquisition, the camera is disconnected from the chamber and covered with a lens cap. Two dark images are captured consecutively under the same exposure time.

**Figure 4:**
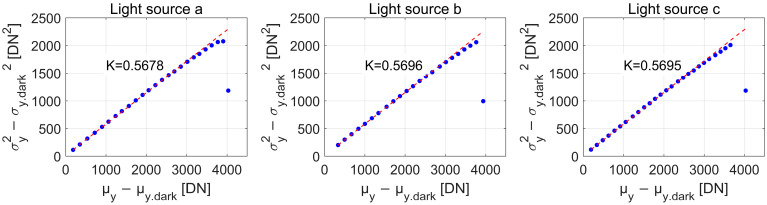
Measurement curves of overall system gain K for Camera B under light sources a − c. Note: All dots represent actual experimental data points. The red dashed line in the background represents the fitted line from the first data point to the highest value point.

The data processing workflow is outlined as follows:

1. Calculation of the mean photo-induced DN $\left(\mu_{y}-\mu_{y.dark}\right)$. The bright-field images are captured at different light sources and saturation levels (30%, 50%, and 90%) for the two cameras. (Note: the saturation level refers to the point where the average gray value of the image reaches the designated percentage of the maximum digital gray value. For instance, in mono12 mode, the maximum gray value is 2^12^ − 1 = 4095, so the 50% saturation level corresponds to approximately 2048. This concept applies to the camera’s sensor response when illuminated by the light sources at different saturation levels.) Specific data for both cameras at these saturation levels are presented in Table 2, derived using Equations (5) and (6).
2. Calculation of the overall system gain K. The noise variance ($\sigma_{y}^{2}-\sigma_{y.dark}^{2}$) is computed using Equations (10) and (11). Based on Equation (9), a linear fit is applied between ($\sigma_{y}^{2}-\sigma_{y.dark}^{2}$) and $\left(\mu_{y}-\mu_{y.dark}\right)$, with the slope representing the system gain K. Figure 4 displays the fitted lines and the corresponding slopes K for Camera B under three different light sources. The calculated data is summarized in Table 2.
3. Calculation of the number of incident photons $\mu_{p}$. Using Equation (14), the total number of incident photons μₚ under various lighting conditions is determined. The spectral integration range spans 400–800 nm, with a discrete interval $\left(\lambda_{i+1}-\lambda_{i}\right)$ of 5 nm. Figure 3 presents the spectral irradiance curve at the image sensor position for Camera B at 50% saturation. The calculated total number of incident photons is 6744, 6803, and 8265 for the respective light sources. Additional results are listed in Table 2.
4. Calculation of the AQE. The AQE is calculated using Equation (4). The AQE results for different lighting conditions are shown in Table 2.

## 4. Discussion and Conclusions

**Figure 5:**
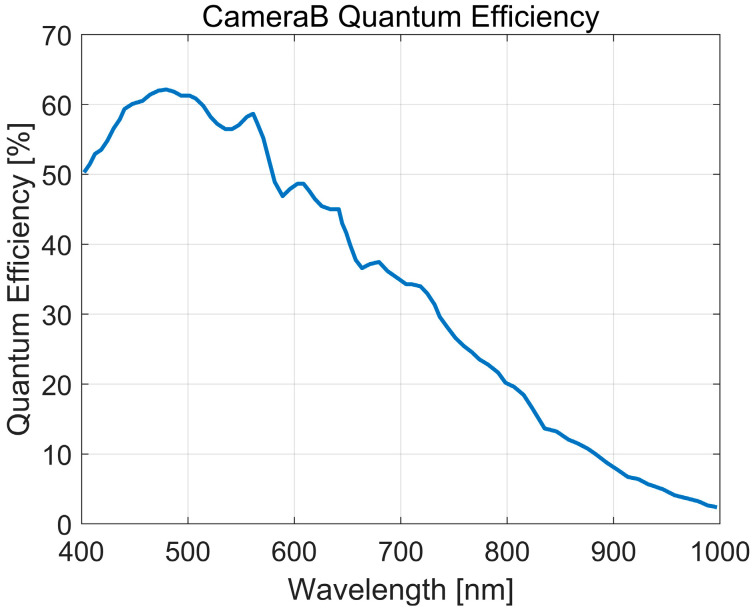
Spectral QE curve of Camera B (derived from the manufacturer’s documentation based on the EMVA1288 standard).

To validate the accuracy of the AQE measurement results, the reference AQE (${\bar{\eta }}_{ref}$) under the experimental light sources was calculated based on the QE curve provided by the manufacturer of Camera B [12]. The spectral QE curve in Figure 5 was redrawn using the QE data from the manufacturer’s measurement protocol for the Basler ace acA2500-14gm, which complies with the EMVA1288 standard. The curve represents QE as a function of wavelength, and its values were used to derive $\eta_{i}$, corresponding to the center wavelength of each interval, as represented on the vertical axis of Figure 5.

At 30%, 50%, and 90% saturation, the reference AQE (${\bar{\eta }}_{ref}$) for the three experimental light sources was calculated with Equations (15) and (16) (see Table 2).

(15) $${\bar{\eta }}_{ref}=\frac{\mu_{e}}{\mu_{p}}=(\sum_{i=1}^{n}\mu_{pi}\eta_{i})/(\sum_{i=1}^{n}\mu_{pi}),$$

(16) $$\mu_{pi}=50.34\cdot A\cdot t_{exp}\cdot \frac{\lambda_{j}+\lambda_{j+1}}{2}\cdot \frac{E_{j}+E_{j+1}}{2}\cdot (\lambda_{j+1}-\lambda_{j}).$$

$\eta_{i}$: QE value corresponding to the center wavelength of the *i*-th interval.
$\mu_{pi}$: Number of photons in the *i*-th interval. The integration range of (400~800) nm is divided into n small intervals with a quantization step of 5 nm, and the photon count for each interval is calculated accordingly. The total number of photons $\mu_{p}=\sum_{i=1}^{n}\mu_{p_{i}}$.
$\mu_{e}$: Total number of electrons, calculated based on the spectral QE curve of the camera and the spectral irradiance distributions of the light sources, $\mu_{e}=\sum_{i=1}^{n}\mu_{pi}\eta_{i}$.

The results show that the AQE measured with light source a closely matches the reference AQE. The value obtained using light source b is approximately 2% higher than the reference value, while the measurement for light source c is about 2% lower. The experimental results are consistent with the reference values, showing the same trend: a > b > c. The discrepancies between the measured and reference AQE values are all within 3%. Considering that the calibration error for photon-related parameters ranges between 3% and 5% (as specified by the EMVA1288 standard [8]), the experimental results presented in this paper are deemed reliable.

The experimental results indicate that the type of light source has no significant impact on the measured system gain K for the same camera, whereas it has a notable effect on the measured AQE. The consistency of the system gain across different light sources can be attributed to its dependence on the internal circuit characteristics of the camera. For the same camera under a given light source, the AQE measurements at different saturation levels exhibit minimal variation. Consequently, AQE measurement can be conducted as soon as the light source stabilizes. Additionally, the performance differences between Camera A and Camera B can be attributed to the distinct sensor technologies used in each camera. Camera A is equipped with the SONY IMX226 back-illuminated sensor, which provides higher QE and better low-light performance. In contrast, Camera B uses a common CMOS sensor, which results in lower quantum efficiency. These differences are reflected in the measured values of system gain (K) and AQE ($\bar{\eta }$) for each camera.

The determination of AQE is strongly influenced by the spectral distribution of the light source and the spectral sensitivity of the camera. A comparison between the spectral distributions of the light sources and the camera’s spectral response curve indicates that the radiation intensity and proportion in the (400–500) nm range follow the order: light source a > b > c. Since the highest sensitivity of digital cameras lies in this range, the highest AQE is obtained with light source a, followed by b, with the lowest AQE measured for light source c.

Mathematically, the AQE calculation can be interpreted as a cross-correlation operation between the spectral distribution function of the light source and the spectral response function of the camera. A closer match between the two functions yields a higher cross-correlation value and, consequently, a higher AQE. For practical applications where the primary concern is to improve the camera’s response in low light conditions, it may be beneficial to select light sources with spectral distributions aligned to the camera’s spectral response function.

This study demonstrates the feasibility of estimating AQE using white light sources, offering a practical method for evaluating camera performance under broadband illumination. This approach is particularly useful for industrial applications with diverse lighting conditions.

## Figures and Tables

**Table 1:** Parameters of the industrial camera under testing.

	Camera A	Camera B
Camera Model	MER2-1220-32U3M	Basler acA2500-14gm
Sensor Type	CMOS Monochrome	
Sensor Chip	SONY IMX226	APTINA MT9P031
Pixel Resolution (W × H)	4024 × 3036	2592 × 1944
Pixel Size	1.85 μm × 1.85 μm	2.2 μm × 2.2 μm
Quantization Depth	12 bits	12 bits
Lens Interface	C-mount	C-mount
Data Interface	USB 3.0	GigE

**Table 2:** AQE measurement results in spectral range 400–800 nm.

	Calculated Parameters	Light Source	30% Saturation	50% Saturation	90% Saturation
Camera A	$\mu_{y}-\mu_{y.dark}$ (DN)	a	1192.7	1987.7	3648.6
		b	1144.5	2037.0	3645.7
		c	1157.4	2093.2	3682.5
	K (DN/e-)	a	0.36		
		b			
		c			
	$\mu_{p}$ (p)	a	4430	7409	13,687
		b	4490	7998	14,440
		c	5192	9354	16,493
	$\bar{\eta }$ (%)	a	75.2	74.9	74.5
		b	70.9	70.9	70.3
		c	62.1	62.3	62.2
Camera B	$\mu_{y}-\mu_{y.dark}$ (DN)	a	1229.8	2050.4	3609.4
		b	1165.9	2028.1	3609.5
		c	1219.1	2050.4	3642.9
	K (DN/e-)	a	0.57		
		b			
		c			
	$\mu_{p}$ (p)	a	4028	6744	11,945
		b	3916	6803	12,169
		c	4939	8265	14,238
	$\bar{\eta }$ (%)	a	53.8	53.5	53.2
		b	52.1	52.1	51.9
		c	43.4	43.6	44.9
	${\bar{\eta }}_{ref}$ (%) for reference	a	53.7	53.4	53.3
		b	50.0	49.9	49.9
		c	46.3	46.4	46.4

## Data Availability

The raw data supporting the conclusions of this article will be made available by the authors on request.
